# Editorial: *Candida* biofilms

**DOI:** 10.3389/fmicb.2022.1128600

**Published:** 2023-01-04

**Authors:** Juliana Campos Junqueira, Eleftherios Mylonakis

**Affiliations:** ^1^Department of Biosciences and Oral Diagnosis, Institute of Science and Technology, São Paulo State University/UNESP, São José dos Campos, Brazil; ^2^Division of Infectious Diseases, Rhode Island Hospital, Alpert Medical School of Brown University, Providence, RI, United States

**Keywords:** biofilms, *Candida* spp., candidiasis, antifungals, fungal-host interactions

Biofilm formation is an important factor of *Candida* pathogenesis with several clinical implications. Most *Candida* spp. form biofilms on mucosal and skin surfaces causing different types of superficial candidiasis, as well as on implanted medical devices leading to systemic infections (Horton et al., 2020; Fan et al., 2022). The inherent biofilm resistance to available antifungal drugs results in recurrent infections, chronic persistent infections, and poor clinical outcomes (Ponde et al., 2021). The Research Topic on “*Candida* biofilms” includes a collection of 14 original research articles and three reviews prepared by renowned groups from Brazil, China, Germany, India, Portugal, Spain, United Kingdom, and USA. Taken together the articles in this issue give an overview on the field of *Candida* biofilms and provide insights on their structure and regulatory networks; interactions with the host immune defense; mechanisms involved in antifungal resistance; pathogenicity and clinical relevance; cross-kingdom interactions; and development of novel therapeutic approaches.

In this context, Böttcher et al. present a detailed study about biofilm formation of *C. albicans* with focus on the role of *STP2*, a key transcriptional regulator of extracellular amino acid signaling and metabolism. The results demonstrate that *STP2* mediates the adherence, germ tube formation, metabolic adaptation, and biofilm sustainability, suggesting that regulatory responses to extracellular amino acids are not only involved with nutritional homeostasis, but also coordinate crucial factors for biofilm development. Related to this work, Wang et al. explore the complex mechanism of *C. albicans* to respond to environmental challenges, unveiling that *SPT20* plays an important role to resist hyperosmotic stress through regulating the high osmolarity glycerol 1 mitogen activated protein kinase transduction pathway (Hog1-MAPK). Moving focus to biofilms formed by *Candida glabrata*, Santos et al. demonstrate that Drug:H+ antiporter 1 (DHA1) transporters, involved in the activation of efflux pumps and drug resistance, can also influence the biofilm development by affecting nutrient uptake and cellular adhesion. Taken together, these studies contribute to clarify the intertwined network of pathways involved in biofilms formed by *Candida* spp., making them promising targets for drug development.

Looking into the influence of biofilms on *Candida*-host interactions and the role of biofilm in *Candida* pathogenesis, Eix and Nett bring a comprehensive review on innate immune responses associated with biofilms, highlighting the key mechanisms by which *Candida* cells increase their resistance to phagocytosis and alter the mononuclear cell cytokine profile. The authors also examine the insights into host responses to biofilm provided by animal studies, and discuss models that explore biofilms formed on vascular catheters, dental devices, and the mucosal surfaces of rats and mice. Using a mouse model of vaginal candidiasis, Wu et al. demonstrate that *C. albicans* strains can form significant quantities of biotic biofilms on the vaginal epithelium. The formation of these biofilms leads to high resistance to antifungal treatment and promotes the formation of persister cells, providing new experimental evidence that extend the role of biofilms in the pathogenesis of vaginal candidiasis. Notably, the mechanisms employed by *C. albicans* to colonize and to form biofilms on vulvovaginal mucosa are thoroughly discussed in the article performed by Rodríguez-Cerdeira et al., who emphasize the genomic, proteomic and quorum sensing aspects of these biofilms.

In a cohort study, Pentland et al. demonstrate the clinical relevance of biofilms in voice prosthesis of patients that underwent total laryngectomy, and show that biofilms are associated with loss of device performance and its early failure. Interestingly, in most cases of prosthesis failure the investigators found polymicrobial biofilms composed mainly by *Staphylococcus aureus* and *C. albicans*. Indeed, multi-species biofilms formed by *Candida* and bacteria can be formed in various niches of the human body, including the oral cavity, gastrointestinal tract, vulvovaginal region, lungs, and skin (Lohse et al., 2018). The interactions established by *Candida* with different bacterial species have been widely studied (Barbosa et al., 2016; Kong et al., 2016; Kostoulias et al., 2016), however little is known about the possible interactions of *Candida* spp. with other fungi. In pioneering studies, Oliveira et al. and Garcia et al. demonstrate that *C. albicans* can form dual species biofilms with *Paracoccidioides brasiliensis* or *Trichophyton rubrum*, respectively. The results of both studies suggest that *C. albicans* and *P. brasilensis* or *T. rubrum* can coexist in the same environment and establish fungal-fungal interactions on host surfaces.

The cross-kingdom microbial interactions in biofilms have been explored as a potential resource for the identification of new antifungal molecules (Scorzoni et al., 2021). From this perspective, Santos et al. reveal that *Streptococcus mutans*, an important bacterium in dental biofilms, can secrete products capable of inhibiting the oral candidiasis in a murine model. In this work, the authors extracted, fractionated, and identified the fraction of the *S. mutans* UA159 culture (SM-F2) with strong activity against *C. albicans* and high efficacy in the treatment of oral candidiasis. In an innovative study, Rossoni et al. explore the antimicrobial activity of bacterial metabolic products on *Candida auris*, an emerging multidrug-resistant yeast. The results show that crude extract derived from the probiotic bacterium *Lactobacillus paracasei* 28.4 can inhibit the biofilms and persister cells of *C. auris*, and protect the model host *Galleria mellonella* from fungal infection through a direct antifungal activity as well as by modulating the host immune response. Besides to natural compounds from microbial origins, plant extracts have gained much attention with large number of bioactive compounds already isolated and identified (Singla and Dubey, 2019; Scorzoni et al., 2021). This Research Topic highlights two plant-derived natural compounds: the coumarin scopoletin (gelseminic acid) studied by Lemos et al. and the palmitic acid (hexadecenoic acid) studied by Prasath et al. Based on their results, both compounds exhibit an effective inhibition on biofilms formed by *Candida tropicalis*. The mechanism of action of scopoletin involves the alteration on fungal cell and plasma membrane sterols, while the action of palmitic acid seems associated with ROS-mediated mitochondrial dysfunction and regulation of ergosterol biosynthesis. Interestingly, scopoletin also showed activity against the efflux pumps at plasma membrane when combined with fluconazole, suggesting potential synergistic activity against multidrug-resistant *Candida* strains.

Looking at therapeutic strategies targeted to *Candida* biofilms, and based on the evidence that antiretroviral HIV protease inhibitors can influence the secreted aspartyl proteases (Saps) of *Candida* spp. (Cenci et al., 2008; Braga-Silva et al., 2010), Lohse et al. investigate the capacity of 80 protease inhibitors in preventing and treating *Candida* biofilms. Among the 80 protease inhibitors studied, the investigators found that gliotoxin, acivicin, TPCK and nelfinavir show effectiveness against *Candida* biofilms. Moreover, several protease inhibitors exhibit ability to decrease *C. albicans* biofilms when combined with caspofungin or amphotericin B. Reddy and Nancharaiah explore new anti-biofilm approaches using ionic liquids, a novel class of molten salts originates from the combination of cations and anions, with several applications in chemical industry. The prospecting results indicate the imidazolium ionic liquid with hexadecyl group ([C16MIM]^+^[Cl]^−^) as the most effective compound against *C. albicans* biofilms. The antifungal and anti-biofilm activity of imidazolium includes alterations in various cellular process, such as membrane permeability, ergosterol content, and ROS generation. Seeking alternative approaches against *C. auris*, Vazquez-Munoz et al. studied the use of silver nanoparticles (AgNPs) coated with polyvinylpyrrolidone (PVP) and verified strong antimicrobial activity on several *C. auris* strains under planktonic and biofilm growing conditions. Promisingly, this antimicrobial activity against *C. auris* strains is irrespective of their clade, geographical origin, or antifungal-resistant profiles.

Lastly, Vera-González and Shukla discuss the recent advances in antifungal biomaterials for combating *Candida* biofilm infections. This review explores the design of nanoparticles aimed at disrupting existing biofilms and presents innovative technologies that employ polymer-only coatings as well as coatings with conventional or new antifungal agents against biofilm formation. Moreover, the authors outline future perspectives in the development of biomaterials targeted for *Candida* biofilms, including the use of enzymes to digest the components of extracellular matrix and identification of new drug targets such as extracellular vesicles.

We hope that this Research Topic covers the key points of the development, pathogenesis, and clinical relevance of *Candida* biofilms, and provides an overview about the progress and challenges of new antifungal discovery that will incentivize innovation in the field of *Candida* biofilm pathogenesis and therapeutics.

## Author contributions

JJ and EM wrote the manuscript. All authors contributed to the article and approved the submitted version.
